# Author Correction: Velocity and density characteristics of subducted oceanic crust and the origin of lower-mantle heterogeneities

**DOI:** 10.1038/s41467-020-16195-8

**Published:** 2020-05-04

**Authors:** Wenzhong Wang, Yinhan Xu, Daoyuan Sun, Sidao Ni, Renata Wentzcovitch, Zhongqing Wu

**Affiliations:** 1 Laboratory of Seismology and Physics of Earth’s Interior, School of Earth and Space Sciences, University of Science and Technology of China, Hefei, China; 2 CAS Center for Excellence in Comparative Planetology, Hefei, China; 3 State Key Laboratory of Geodesy and Earth’s Dynamics, Institute of Geodesy and Geophysics, Chinese Academy of Sciences, 430077 Wuhan, China; 4 Department of Applied Physics and Applied Mathematics, Columbia University, New York, NY 10027 USA; 5 Department of Earth and Environmental Sciences, Columbia University, New York, NY 10027 USA; 6 Lamont–Doherty Earth Observatory, Columbia University, Palisades, NY 10964 USA

**Keywords:** Geodynamics, Geophysics, Mineralogy

Correction to: *Nature Communications* 10.1038/s41467-019-13720-2, published online 7 January 2020.

The original version of this Article omitted the following from the Acknowledgements:

R.M.W. was supported by National Science Foundation award EAR-1918126 and by the US Department of Energy award DESC0019759.

This has now been corrected in both the PDF and HTML versions of the Article.

