# Supplementary material for: Velocity and density characteristics of subducted oceanic crust and the origin of lower-mantle heterogeneities
Source: Nat Commun. 2020 Jan 7;11:64. doi: 10.1038/s41467-019-13720-2 (PMC6946644; doi:10.1038/s41467-019-13720-2)
Supplement: Supplementary file 1 — Supplementary Information [file 41467_2019_13720_MOESM1_ESM.pdf]

Supplementary Information for “Velocity and density characteristics of subducted oceanic crust and the origin of lower-mantle heterogeneity” by Wang et al.

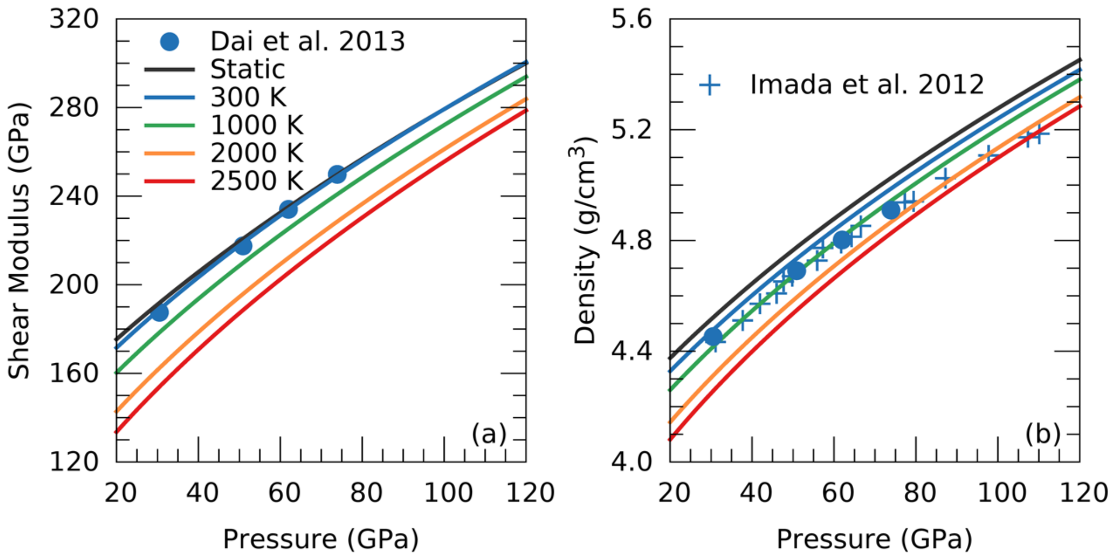

Supplementary Figure 1. Shear modulus (G) and density (ρ) of CF-type phase with the chemical composition of $Na_{0.4}Mg_{0.6}Al_{1.6}Si_{0.4}O_4$ compared with experimental measurements. The G and ρ of $Na_{0.4}Mg_{0.6}Al_{1.6}Si_{0.4}O_4$ are calculated from those of two end-members ($NaAlSiO_4$ and $MgAl_2O_4$) using the interpolation method. Experimental data are from Dai et al. (2013)(*1*) and Imada et al. (2012)(*2*). The calculated density of $Na_{0.4}Mg_{0.6}Al_{1.6}Si_{0.4}O_4$ CF-type phase agree well with experimental data in Imada et al. (2012) at low pressures but deviates from experimental measurements when pressure is higher than 80 GPa. This is probably due to the large non-hydrostatic pressure at high pressure since the high-pressure runs of Imada et al. (2012) were conducted at the lack of pressure medium.

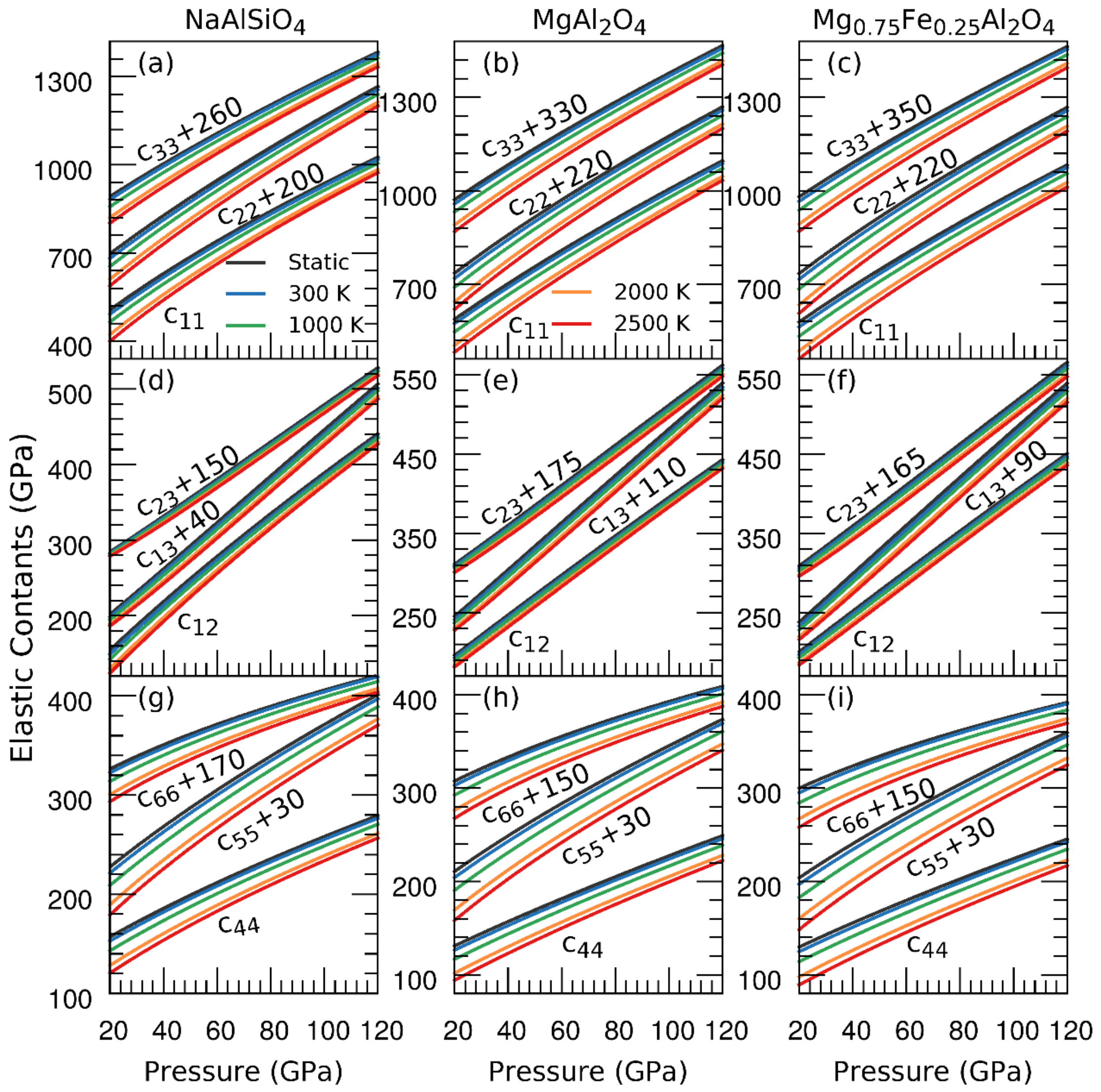

Supplementary Figure 2. Elastic constants of CF-type phae at variable pressures and temperatures. (a) (d) (g) $NaAlSiO_4$, (b) (e) (h) $MgAl_2O_4$, and (c) (f) (i) $Mg_{0.75}Fe_{0.25}Al_2O_4$).

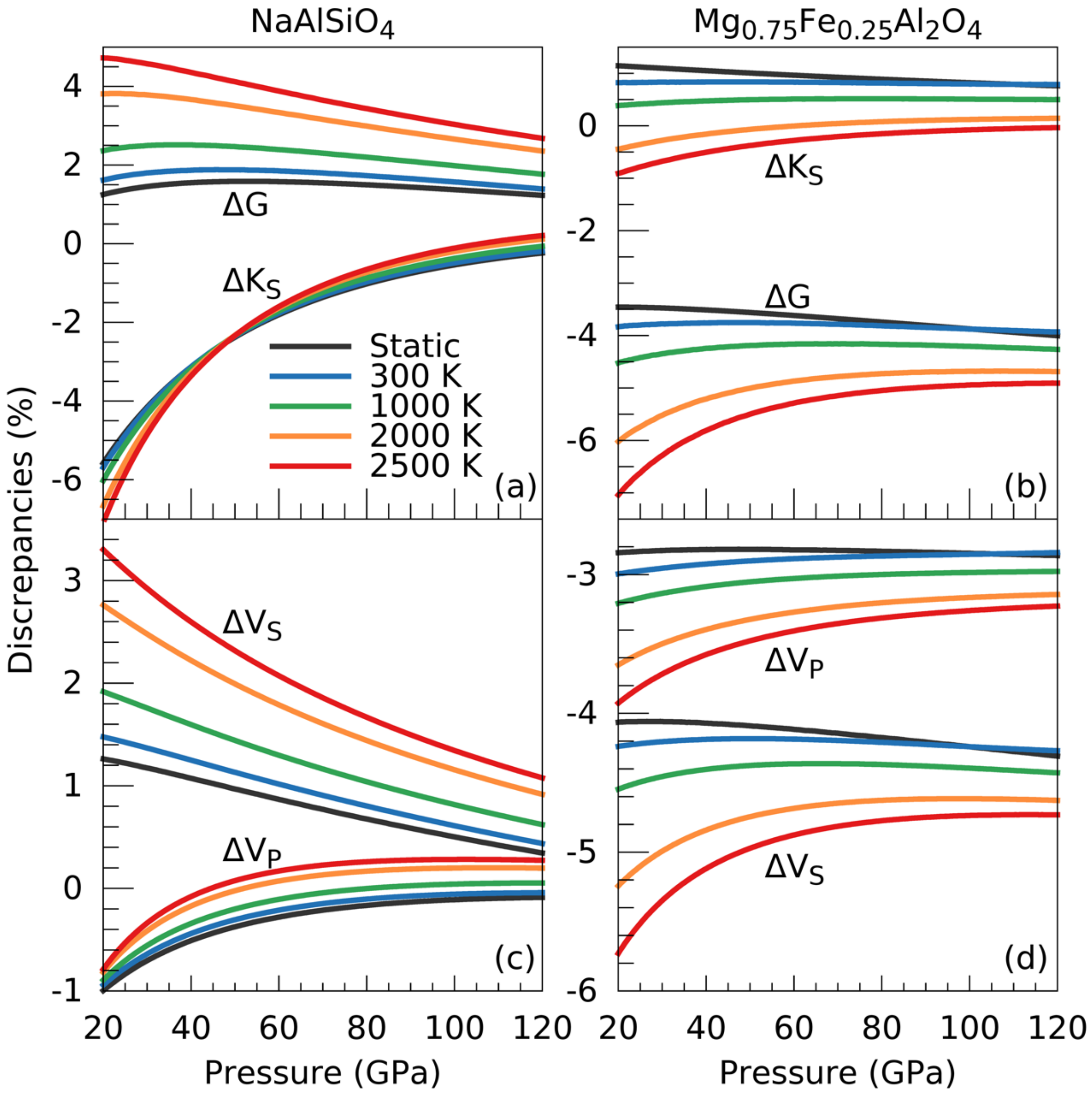

Supplementary Figure 3. Relative discrepancies in elastic moduli and velocities of CF-type phase. (a) (b) bulk and shear moduli (Ks and G), (c) (d) compressional and shear wave velocities (V_P and V_S). We take these values of $MgAl_2O_4$ CF-type phase as references.

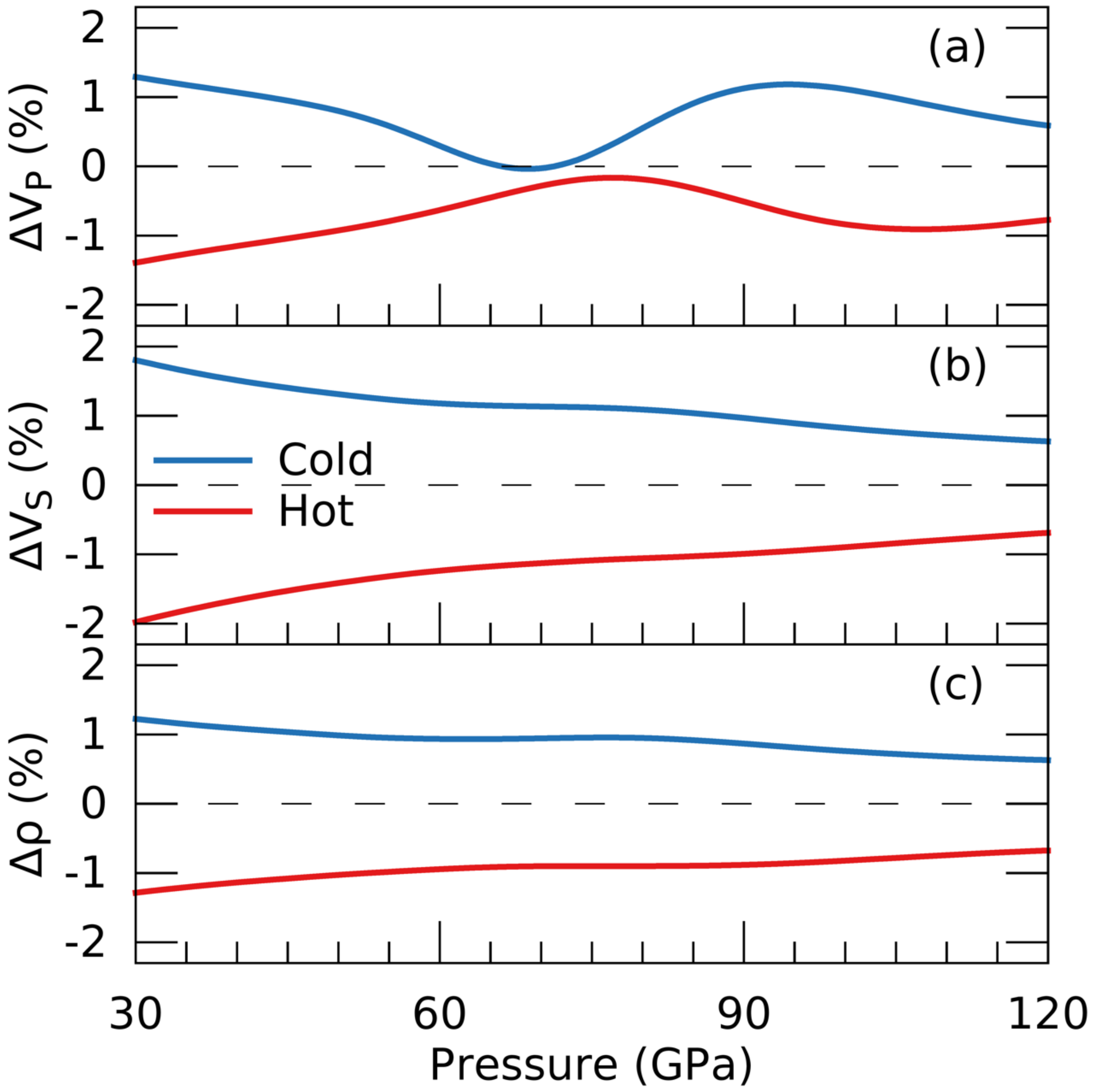

Supplementary Figure 4. Velocity and density perturbations for the pyrolitic composition caused by the temperature variation. (a) compressional (b) shear wave velocity perturbations (ΔV_P and ΔV_S); (c) density perturbation ($\Delta\rho$). The pyrolitic lower mantle(*3*) adopted here is composed of 15% ferropericlase ($Mg_{0.82}Fe_{0.18}O$), 78% Fe-bearing bridgmanite ($Mg_{0.92}Fe_{0.08}SiO_3$), and 7% Ca-perovskite ($CaSiO_3$). The velocity and density of pyrolite model along the normal mantle geotherm(*4*) are taken as references. Temperatures along hot and cold geotherms are assumed to be 500 K higher and lower than that of normal mantle geotherm, respectively.

Supplementary Table 1. Bulk moduli (Ks), shear moduli (G), compressional wave velocities (V_P), and shear wave velocities (V_S) of $NaAlSiO_4$, $MgAl_2O_4$, and $Mg_{0.75}Fe_{0.25}Al_2O_4$ CF-type phases. The units of moduli and velocities are gigapascal (GPa) and kilometer per second (km/s), respectively.

T (K)	P (GPa)	$NaAlSiO_4$				$MgAl_2O_4$				$Mg_{0.75}Fe_{0.25}Al_2O_4$			
		Ks	G	V_P	V_S	Ks	G	V_P	V_S	Ks	G	V_P	V_S
300	30	313.8	191.3	11.273	6.537	327.5	185.9	11.346	6.449	330.2	179.2	11.010	6.178
	40	353.0	206.7	11.674	6.694	364.4	201.1	11.725	6.612	367.4	193.9	11.382	6.335
	60	428.4	234.6	12.354	6.950	436.2	229.0	12.380	6.880	439.8	220.7	12.023	6.592
	80	500.6	259.5	12.920	7.153	505.5	254.2	12.933	7.096	509.6	244.7	12.562	6.797
	100	570.0	282.1	13.405	7.320	572.8	277.4	13.412	7.275	577.4	266.8	13.029	6.967
1000	30	302.9	181.6	11.107	6.411	316.6	175.0	11.169	6.301	318.0	167.7	10.819	6.020
	40	342.8	197.7	11.532	6.585	354.0	190.9	11.571	6.481	355.7	183.1	11.214	6.196
	60	419.5	226.6	12.247	6.863	426.8	219.9	12.260	6.775	428.9	211.0	11.888	6.480
	80	492.7	252.3	12.836	7.081	497.0	245.9	12.837	7.009	499.6	235.9	12.452	6.702
	100	563.1	275.6	13.339	7.260	565.1	269.7	13.334	7.201	568.0	258.5	12.936	6.885
2000	30	285.2	166.6	10.841	6.212	299.1	158.2	10.886	6.062	298.3	149.6	10.505	5.759
	40	326.0	183.9	11.306	6.414	337.1	175.2	11.326	6.275	336.6	166.3	10.941	5.971
	60	404.4	214.5	12.078	6.732	411.1	205.9	12.070	6.614	411.1	196.1	11.675	6.304
	80	479.2	241.5	12.707	6.976	482.7	233.3	12.686	6.877	483.1	222.4	12.280	6.559
	100	550.8	265.8	13.239	7.173	552.0	258.2	13.212	7.091	552.6	246.2	12.794	6.764
2500	30	276.0	158.8	10.694	6.102	290.1	149.3	10.731	5.928	288.1	140.1	10.332	5.611
	40	317.3	176.6	11.183	6.321	328.4	167.0	11.192	6.161	326.7	157.5	10.792	5.846
	60	396.6	208.2	11.988	6.662	403.0	198.6	11.968	6.527	401.9	188.4	11.560	6.208
	80	472.1	235.9	12.638	6.921	475.2	226.7	12.605	6.807	474.4	215.5	12.188	6.482
	100	544.4	260.8	13.185	7.128	545.0	252.2	13.148	7.033	544.6	239.9	12.719	6.700

Supplementary References

1. L. Dai *et al.*, Sound velocities of $Na_{0.4}Mg_{0.6}Al_{1.6}Si_{0.4}O_4$ NAL and CF phases to 73 GPa determined by Brillouin scattering method. *Phys. Chem. Miner.* **40**, 195–201 (2013).
2. S. Imada, K. Hirose, T. Komabayashi, T. Suzuki, Y. Ohishi, Compression of $Na_{0.4}Mg_{0.6}Al_{1.6}Si_{0.4}O_4$ NAL and Ca-ferrite-type phases. *Phys. Chem. Miner.* **39**, 525–530 (2012).
3. Z. Wu, Velocity structure and composition of the lower mantle with spin crossover in ferropericlase. *J. Geophys. Res. Solid Earth*. **121**, 2304–2314 (2016).
4. J. M. Brown, T. J. Shankland, Thermodynamic parameters in the Earth as determined from seismic profiles. *Geophys. J. Int.* **66**, 579–596 (1981).
